# Serological detection of *Anaplasma phagocytophilum*, *Borrelia burgdorferi* sensu lato and *Ehrlichia canis* antibodies and *Dirofilaria immitis* antigen in a countrywide survey in dogs in Poland

**DOI:** 10.1007/s00436-014-3985-7

**Published:** 2014-06-29

**Authors:** Friederike Krämer, Roland Schaper, Bettina Schunack, Andrzej Połozowski, Jolanta Piekarska, Aleksandra Szwedko, Robert Jodies, Dagmara Kowalska, Dörte Schüpbach, Nikola Pantchev

**Affiliations:** 1 Institute for Parasitology and Tropical Veterinary Medicine, Faculty of Veterinary Medicine, Freie Universität Berlin, 14163 Berlin, Germany; 2 Bayer Animal Health GmbH, 51373 Leverkusen, Germany; 3 Department of Internal Medicine and Clinic of Horses, Dogs and Cats, Faculty of Veterinary Medicine, Wroclaw University of Environmental and Life Sciences, 50-366 Wroclaw, Poland; 4 PPH Eskulap, 44-105 Gliwice, Poland; 5 Lekdijk Oost 11, 4112PB Beusichem, The Netherlands; 6 Bayer Sp. z o.o., Animal Health Division, 02-326 Warsaw, Poland; 7 IDEXX Vet Med Labor GmbH, 71636 Ludwigsburg, Germany

**Keywords:** Dog, Canine vector-borne diseases (CVBDs), SNAP® 4Dx®, Prevalence, Distribution, Poland

## Abstract

Canine vector-borne diseases (CVBDs) have increasingly become a focus of attention in the past few years. Nevertheless, in many parts of Europe information on their occurrence is still scarce. In a large study in Poland 3,094 serum samples taken from dogs throughout all 16 Polish provinces were tested using a commercial kit for the detection of circulating antibodies against *Anaplasma phagocytophilum*, *Borrelia burgdorferi* sensu lato and *Ehrlichia canis* and of *Dirofilaria immitis* antigen. A total of 12.31 % (381/3,094; 95 % confidence interval [CI]: 11.18–13.52 %) and 3.75 % (116/3,094; 95 % CI: 3.11–4.48 %) of the dogs were positive for *A. phagocytophilum* and *B. burgdorferi* s.l. antibodies, respectively. Furthermore, 0.26 % (8/3,094; 95 % CI: 0.11–0.51 %) were positive for *E. canis* antibodies and 0.16 % (5/3,094; 95 % CI: 0.05–0.38 %) for *D. immitis* antigen. The highest percentages of *A. phagocytophilum*-positive dogs were noted in Lesser Poland, Silesia and Łódź Provinces. For *B. burgdorferi* s.l., the highest prevalence was recorded in Łódź Province. Co-infections with *A. phagocytophilum* and *B. burgdorferi* s.l. were recorded in 1.71 % of all examined dogs (53/3,094; 95 % CI: 1.29–2.23 %). One dog even had a triple infection, testing positive for *E. canis* too. Both *A. phagocytophilum* and *B. burgdorferi* s.l. have previously been reported in Poland and were confirmed in the present study by positive samples from all 16 provinces. Concerning *E. canis* and *D. immitis* travel history or importation cannot be excluded as factors which may have determined the occurrence of these pathogens in the relevant animals. Practitioners in Poland should be aware of the above mentioned CVBDs and of prophylactic measures to protect dogs and their owners.

## Introduction

Canine vector-borne diseases (CVBDs) have increasingly become a focus of interest in recent years. Long-term climate change on the one hand, and biotic factors — such as an increase in reservoir abundance, changing habitat structure, socio-political changes and, especially for dogs, increasing travel and dog import for welfare reasons — on the other hand are discussed in this context as parameters for the expansion of vectors and pathogens into formerly unaffected areas. Two of these canine vector-borne pathogens, *Anaplasma phagocytophilum* and *Borrelia burgdorferi* sensu lato, have now been reported in dogs in nearly all European countries. In some countries, the pathogens have only been reported in the vector, e.g., *A. phagocytophilum* in *Ixodes ricinus* ticks in Finland (unpublished data by E. Hasu cited in Heikkilä et al. 2010), Estonia (Katargina et al. 2012) and Lithuania (Paulauskas et al. 2012), or in animals other than dogs, e.g., in a cat in Finland (Heikkilä et al. 2010), but data on canine prevalence of *A. phagocytophilum* have yet to be published. From the Baltic States plus Belarus, for example, a canine study with confirmed occurrence of *A. phagocytophilum* could be found only for Latvia (Bērziņa and Matīse 2013). Other studies screened only small canine populations in a restricted focus.

The highest number of human cases of borreliosis in Poland in 2011 was registered in Podlaskie Province with 75.5 per 100,000 people (Paradowska-Stankiewicz and Chrześcijańska 2013). Most cases of borreliosis in Poland originally occurred in this north-eastern region, but the disease is no longer solely a problem in this part of the country (Paradowska-Stankiewicz and Chrześcijańska 2013). Other studies on ticks and forest workers in the north-western part of the country revealed prevalences between 7.4 % (Skotarczak et al. 2002) and 16.7 % (Skotarczak et al. 2003) in the tick population and 61 % in forest workers (Niścigorska et al. 2003). Seropositivity was also recorded in dogs in the north-western part of Poland (Skotarczak and Wodecka 2003, 2005). The main vector in the area for the pathogen *B. burgdorferi* s.l. is *I. ricinus*, which is generally distributed throughout the country.

The pathogen *A. phagocytophilum* is reported to occur in its vector *I. ricinus* in numerous studies in Poland. The prevalence in ticks has been reported across the country (from the north-west (Rymaszewska 2005) to the south-east (Cisak et al. 2005)), ranging from 2.9 % in the central region (Warsaw) (Zygner et al. 2008) to 76.7 % in the south (Lesser Poland) (Asman et al. 2013). In man (mainly forest workers as an especially tick-exposed group within the population), antibodies against *A. phagocytophilum* have also been detected, e.g., in 17.7 % in north-eastern Poland (Roztocze National Park in Lublin) (Cisak et al. 2005) and 19.8 % in the Lublin region (Zwoliński et al. 2004). Finally, dogs have been screened in a few studies, with 2/192 dogs being seropositive for *A. phagocytophilum* in north-western Poland (Skotarczak et al. 2004), 14 % of dogs suspected of having Lyme disease being positive for *A. phagocytophilum* in a study from Szczecin University (Rymaszewska and Adamska 2011), and 1/79 dogs being positive in a group of apparently healthy sled dogs (Welc-Falęciak et al. 2009). In addition to the occurrence of the pathogen in dogs, it is also reported in diverse forms of wild life in Poland (e.g., roe deer (Welc-Falęciak et al. 2013); wild boars (Michalik et al. 2012); wild cervids (Hapunik et al. 2011)). Even though this aspect is not examined very often within the canine population, there is a clear risk of infection by frequently reported *A. phagocytophilum*-positive *I. ricinus* ticks in Poland.

Autochthonous cases of *Ehrlichia canis* have so far not been reported in dogs in Poland, and for *Dirofilaria immitis* only one questionable autochthonous case in Poland without molecular confirmation was described (Światalska and Demiaszkiewicz 2012).

The vector for *E. canis* is *Rhipicephalus sanguineus* (Groves et al. 1975; Lewis et al. 1977), which in Europe mainly occurs in places with a Mediterranean climate. For Poland, only two citations of *R. sanguineus* occurrence could be found. One on a mass infestation in an apartment in Warsaw in the 1970s (Szymański 1979) and one on a dog in Warsaw, which might be identical with the publication of the mass infestation in the 1970s, as it is only mentioned in a review by Nowak-Chmura and Siuda (2012) without concrete citation. As Poland is not endemic for *R. sanguineus*, it can be suggested that *E. canis* infection is not autochthonously occurring in Poland, but is associated with import of dogs or a travel history. Exceptionally, imported ticks may establish populations within all-year temperate homes and subsequently lead to an “autochthonous” infection, as suggested for Germany (Dongus et al. 1996).

Apart from the *D. immitis* case mentioned above, *Dirofilaria repens* has so far been detected only in dogs in central Poland (Demiaszkiewicz et al. 2009) and in dogs imported from Poland (Pantchev et al. 2011). Typical endemic areas for *D. immitis* are found in the Mediterranean region extending up to the Alps. As far as the countries bordering on Poland are concerned, individual cases have been detected in Slovakia (Iglódyová et al. 2012). Nevertheless, evaluating the temperature records, spanning a 29-year period (1971–2000), along with the model of Fortin and Slocombe (1981) modified by Lok and Knight (1998), for eastern Europe, a threshold value of 130 cumulative *Dirofilaria* developing units (DDU) reached in 30 consecutive days, being sufficient to facilitate extrinsic incubation of *Dirofilaria*, were also recorded for Poland between June and August and to a very reduced amount as well in September (Genchi et al. 2011).

The aim of the study described here was to collect current data on the occurrence and distribution of four major canine vector-borne pathogens via a large nationwide survey of the canine population in Poland. A further aim was to characterise in more detail mixed infections with the various pathogens and areas of high prevalence.

## Material and methods

### General

Serum samples from 3,094 dogs were analysed in the study. The samples were taken by local veterinarians in 54 participating veterinary practices distributed throughout all 16 Polish provinces. The practices were participating in a research project which formed part of the “European Project for *Anaplasma* and *Borrelia* Prevalence in Dogs”. The samples were submitted to a diagnostic laboratory for analysis. The origin of the dogs was determined using the postcode supplied with the sample.

### Clinical samples, study period, study area

The serum was collected from clinically healthy dogs with a tick history visiting veterinary practices in all 16 Polish provinces. The samples were collected between March and October 2011.

### Laboratory tests, data calculation and visualisation

**Fig. 1:**
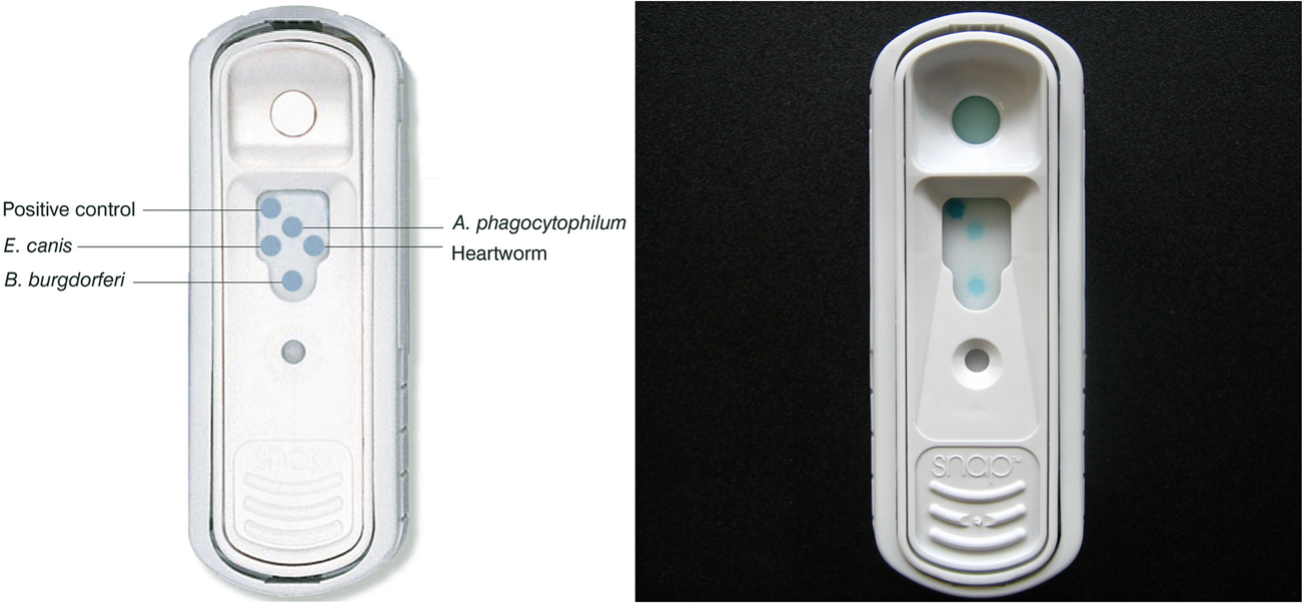
Canine SNAP® 4Dx® test device. Schematic illustration (*left*) and photograph of a test device demonstrated with a canine serum sample positive for *Borrelia* and *Anaplasma* antibodies (*right*)

The samples were picked up from veterinary clinics by PPH Eskulap, Gliwice, and then submitted to a private veterinary diagnostic laboratory (IDEXX Vet Med Lab, Ludwigsburg, Germany) for testing of different CVBDs. Serological testing was performed using a rapid assay test system (SNAP® 4Dx®, IDEXX Laboratories, Inc., Westbrook, ME, USA) following the manufacturer’s instructions for use. SNAP® 4Dx® (Fig. 1) is a rapid assay test system based on enzyme immunoassay technique. The test has been validated for dogs (Chandrashekar et al. 2010) and is officially registered for use in dogs in Germany by the Friedrich Loeffler Institute (FLI). A test unit consists of a coated membrane matrix with five spots in the reaction area (result window). Three spots are impregnated respectively with a specific peptide antigen of *A. phagocytophilum* (a synthetic peptide from the major surface protein (p44/MSP2)), *B. burgdorferi* s.l. (C6 peptide) and *E. canis* (peptides from p30 and p30-1 outer membrane proteins). The *D. immitis* analyte is derived from antibodies specific to heartworm antigens, which are primarily produced by adult females (Weil 1987). The fifth spot serves as a positive control. A two-chamber system contains wash solution and substrate solution, which flow across the coated membrane upon activation of the test (Pantchev et al. 2009a). The sensitivity of the performed test ranges according to the manufacturer from 99.1 % for *A. phagocytophilum* and 98.8 % for *B. burgdorferi* s.l. to 96.2 % for *E. canis* and 99.2 % for *D. immitis*, with a specificity for all four pathogens of 100 % according to Chandrashekar and colleagues (2010). Antibodies against *Anaplasma platys* in experimentally infected dogs have cross-reacted with the *A. phagocytophilum* analyte, and the *E. canis* analyte may cross-react with anti-*Ehrlichia chaffeensis* antibodies (Chandrashekar et al. 2010). Nevertheless, one natural *A. platys* infection in a dog yielded a negative result in this test (Dyachenko et al. 2012). Cross-reactivity of the *D. immitis* analyte in similar commercially available antigen tests with *Angiostrongylus vasorum*-positive dogs has since been described (Schnyder and Deplazes 2012) and will be discussed later in this publication.

There are a number of different genospecies concerning *B. burgdorferi* s.l. There are at least three species in Europe that are pathogenic for humans: *B. burgdorferi* sensu stricto, *B. garinii* and *B. afzelii*. The C6 peptide is antigenically conserved among them and may be used to serodiagnose borreliosis universally (Liang et al. 2000). Detection of antibodies against C6 peptide in dogs by means of commercially available tests does not interact with *Borrelia* vaccination (O'Connor et al. 2004), succeeds from days 21 to 35 post-infection onwards (Wagner et al. 2012), and persists in untreated dogs for at least 12 months (Levy et al. 2008).

The collected data were analysed by a geographic information system (GIS) using the programme RegioGraph 10 (GfK GeoMarketing, Bruchsal, Germany) to visualise the regional distribution of collected and analysed serum samples and antibody- and/or antigen-positive samples for the different pathogens on administrative maps. Using the three digits as points of reference, the locations of positive samples were displayed on maps with administrative and postcode boundaries.

The descriptive analysis was performed with the help of the validated statistical programme TESTIMATE Version 6.5 from IDV Data Analysis and Study Planning. The presence of antibodies (for *A. phagocytophilum*, *B. burgdorferi* s.l. and *E. canis*) or antigen (for *D. immitis*) for every variable was dichotomised into negative (=no presence) and positive (=presence) to calculate the prevalence and the 95 % confidence interval (CI). Additionally, differences from the overall sum for each of the 16 provinces were calculated using the Fligner-Wolfe test (many-to-many test, alpha = 0.05) for *A. phagocytophilum*, *B. burgdorferi* s.l. and co-infection with *A. phagocytophilum* and *B. burgdorferi* s.l.

## Results

The seropositivity of all tested samples is summarised in Table 1. The overall prevalence of *A. phagocytophilum* and *B. burgdorferi* s.l. in dogs was 12.31 % (*n* = 381; 95 % CI: 11.18–13.52 %) and 3.75 % (*n* = 116; 95 % CI: 3.11–4.48 %), respectively. The overall prevalence based on the test results for *E. canis* and *D. immitis* in dogs was 0.26 % (*n* = 8; 95 % CI: 0.11–0.51 %) and 0.16 % (*n* = 5; 95 % CI: 0.05–0.38 %), respectively.

**Table 1:** Results of dog serum samples from Poland (*n* = 3,094) tested for the presence of specific antibodies against *Anaplasma phagocytophilum* (Ap), *Borrelia burgdorferi* s.l. (Bb) and *Ehrlichia canis* (Ec) and of circulating antigen of *Dirofilaria immitis* (Di)

Causative organism	Antibody (Ap, Bb, Ec) or antigen (Di) positive dogs/all tested dogs	Percentage	95 % Confidence interval
*Anaplasma phagocytophilum*	381/3,094	12.31 %	11.18–13.52 %
*Borrelia burgdorferi* s.l.	116/3,094	3.75 %	3.11–4.48 %
*Ehrlichia canis*	8/3,094	0.26 %	0.11–0.51 %
*Dirofilaria immitis*	5/3,094	0.16 %	0.05–0.38 %

The results of the *D. immitis* test component of this study need to be discussed differentially. Simultaneous use of highly specific diagnostic methods to differentiate “true” canine heartworm (*D. immitis*) and “French” heartworm (*A. vasorum*, a potentially fatal canine nematode that also lives as an adult in the pulmonary arteries) is recommended within overlapping endemic areas, as some commercially available heartworm antigen tests show cross-reactivity with *A. vasorum* (Schnyder and Deplazes 2012). Nowadays, a revised version of the test system used in this study, SNAP® 4Dx® Plus (IDEXX Laboratories, Inc., Westbrook, ME, USA), which does not show any cross-reactivity between *D. immitis* and *A. vasorum* (Schnyder and Deplazes 2012), and a specific rapid *A. vasorum* device (Schnyder et al. 2014) are available, but they were not on the market when testing was performed for the present study. Nevertheless, it was possible to follow the above recommendation of simultaneously using highly specific diagnostic methods as the dog population in the study reported here was partially identical with that in a study that examined dogs for the presence of *A. vasorum* antibodies and antigen in Poland (Schnyder et al. 2013). The results of the two studies were compared.

Looking at the five *D. immitis*-positive dogs in this study, a positive *A. vasorum* antigen sandwich ELISA (Schnyder et al. 2011) and *A. vasorum* antibody sandwich ELISA (Schucan et al. 2012) were reported for one dog from Masovia Province, pointing to potential cross-reactivity between *D. immitis* and *A. vasorum*. No further information for this dog was available, in particular regarding possible travel to *D. immitis*-endemic areas. If this dog needed to be considered in terms of a potential cross-reaction with *A. vasorum*, which would be the case if it had no history of travelling abroad, the prevalence of *D. immitis* would have to be corrected to 0.13 % (4/3094; 95 % CI: 0.04–0.33 %). The other four dogs (from Warmia-Masuria, Opole, Greater Poland and Pomerania Provinces) showed an optical density in the *A. vasorum* antigen sandwich ELISA well below the cut-off within the tested population of Polish dogs (Schnyder et al. 2013) and were thus classified as *A. vasorum*-negative in the corresponding study. A negative *A. vasorum* antibody ELISA for these dogs in Schnyder and colleagues (2013) confirms an *A. vasorum*-negative status and thus a true *D. immitis*-positive result in the performed SNAP® 4Dx® test.

**Fig. 2:**
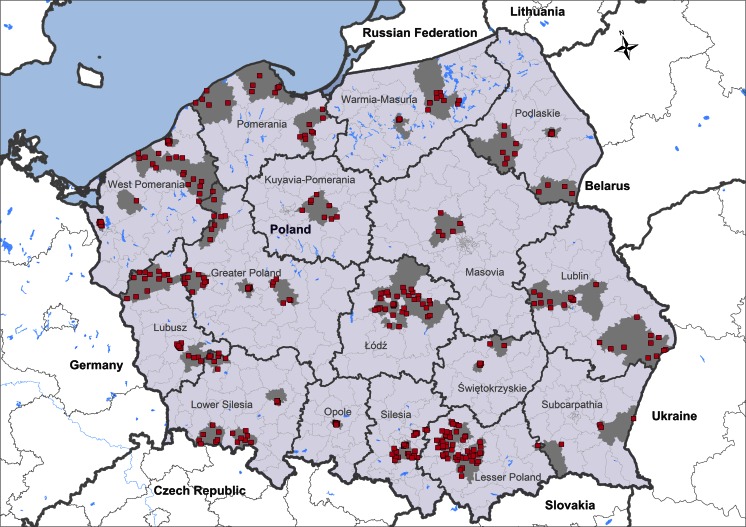
Occurrence of *Anaplasma phagocytophilum*-positive dogs detected by SNAP® 4Dx® in a population of 3,094 from Poland. *Dark grey areas* represent the origin of the tested dog sera. The origins of dogs positive for circulating *A. phagocytophilum* antibodies (*n* = 381) are shown in *red*

**Fig. 3:**
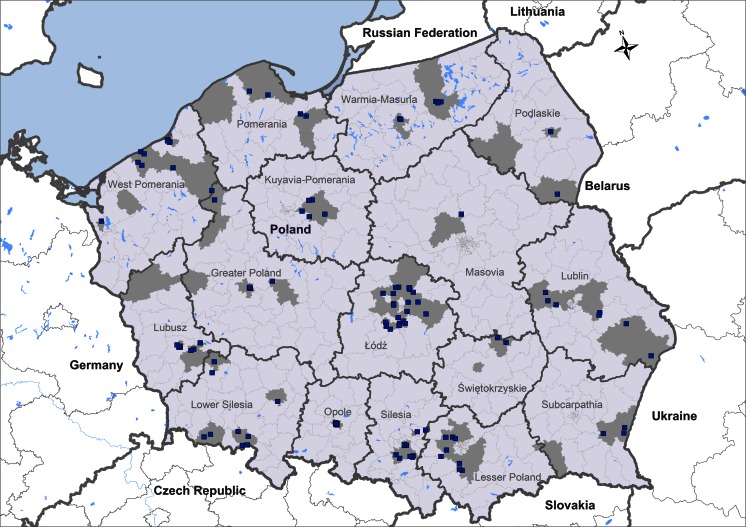
Occurrence of *Borrelia burgdorferi* s.l. -positive dogs detected by SNAP® 4Dx® in a population of 3,094 from Poland. *Dark grey areas* represent the origin of the tested dog sera. The origins of dogs positive for circulating *B. burgdorferi* s.l. antibodies (*n* = 116) are shown in *blue*

**Fig. 4:**
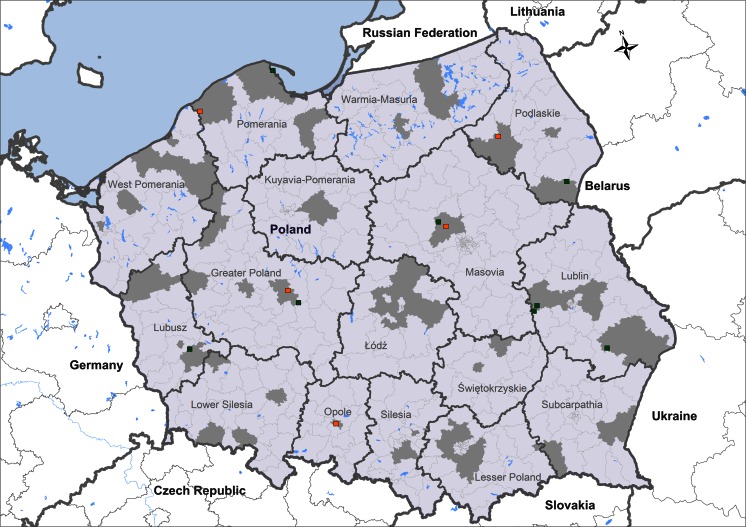
Occurrence of *Ehrlichia canis-* and *Dirofilaria immitis*-positive dogs detected by SNAP® 4Dx® in a population of 3,094 from Poland. *Dark grey areas* represent the origin of the tested dog sera. The origins of dogs positive for circulating *E. canis* antibodies (*n* = 8) and *D. immitis* antigen (*n* = 5) are shown in *green* and *orange*, respectively

The locations of the positive samples (as coloured spots) and the sampling area (in dark grey) on the administrative maps are shown in Figs. 2, 3 and 4. Furthermore, the number of positive samples per province is shown in Table 2.

**Table 2:** Distribution of *Anaplasma phagocytophilum* (Ap), *Borrelia burgdorferi* s.l. (Bb), *Ehrlichia canis* (Ec) and *Dirofilaria immitis* (Di) positive samples per province (percentage and total numbers)

Province (number of veterinary clinics)	Percentage Ap positive (*x*/*y*)	Percentage Bb positive (*x*/*y*)	Percentage Ec positive (*x*/*y*)	Percentage Di positive (*x*/*y*)	Percentage Ap + Bb positive (*x*/*y*)	Percentage Ap + Bb + Ec positive (*x*/*y*)
Greater Poland (5)	12.72 % (43/338)	2.66 % (9/338)	0.30 % (1/338)	0.30 % (1/338)	0.89 % (3/338)	
Kuyavia-Pomerania (1)	10.26 % (8/78)	6.41 % (5/78)			1.28%c (1/78)	
Lesser Poland (4)	25.24 % (53/210)	3.81 % (8/210)			2.86 % (6/210)	
Łódź (4)	20.28 % (43/212)	11.32 % (24/212)			8.49 % (18/212)	
Lower Silesia (3)	13.39 % (15/112)	5.36 % (6/112)			1.79 % (2/112)	
Lublin (5)	7.50 % (24/320)	2.19 % (7/320)	0.94 % (3/320)		0.94 % (3/320)	
Lubusz (3)	16.49 % (32/194)	4.64 % (9/194)	0.52 % (1/194)		1.55 % (3/194)	0.52 % (1/194)
Masovia (2)	3.21 % (5/156)	0.64 % (1/156)	0.64 % (1/156)	0.64 % (1/156)/ 0.0 % (0/156)^a^		
Opole (1)	15.00 % (18/120)	6.67 % (8/120)		0.83 % (1/120)	1.67 % (2/120)	
Podlaskie (2)	9.68 % (12/124)	1.61 % (2/124)	0.81 % (1/124)			
Pomerania (6)	8.23 % (20/243)	1.65 % (4/243)	0.41 % (1/243)	0.41 % (1/243)	0.82 % (2/243)	
Silesia (3)	20.90 % (42/201)	5.97 % (12/201)			3.48 % (7/201)	
Subcarpathia (2)	4.27 % (5/117)	2.56 % (3/117)				
Świętokrzyskie (2)	4.80 % (6/125)	1.60 % (2/125)			0.80 % (1/125)	
Warmia-Masuria (5)	8.73 % (20/229)	2.62 % (6/229)		0.44 % (1/229)	0.87 % (2/229)	
West Pomerania (6)	11.11 % (35/315)	3.17 % (10/315)			0.95 % (3/315)	
Total (54)	12.31 % (381/3,094)	3.75 % (116/3,094)	0.26 % (8/3,094)	0.16 % (5/3,094)/0.13 % (4/3,094)^a^	1.71 % (53/3,094)	0.03 % (1/3,094)

*x* samples positive for a specific pathogen, *y* total number of samples tested per province

^a^Corrected data after discounting one potential *A. vasorum* cross-reacting dog in Masovia Province

Co-infections with *A. phagocytophilum* and *B. burgdorferi* s.l. were observed in 1.71 % (*n* = 53; 95 % CI: 1.29–2.23 %) of the tested dogs. One dog proved to have a triple infection with *A. phagocytophilum*, *B. burgdorferi* s.l. and *E. canis*. The proportion of single, double and triple infections in the sum of all positive samples is listed in Table 3.

**Table 3:** Proportion of single, double and triple infections in positive samples (*n* = 456)

	Ap (alone)	Bb (alone)	Ec (alone)	Di	Ap + Bb	Ap + Bb + Ec
Positive samples	328	63	7	5	52	1
Percentage (95 % CI)	71.93 % (67.56–76.01 %)	13.82 % (10.78–17.33 %)	1.54 % (0.62–3.14 %)	1.10 % (0.36–2.54 %)	11.40 % (8.63–14.68 %)	0.22 % (0.01–1.22 %)

Ap* Anaplasma phagocytophilum*, Bb* Borrelia burgdorferi* s.l., Di* Dirofilaria immitis*, Ec* Ehrlichia canis*, CI confidence interval

A travel history or importation cannot be excluded for dogs positive for *E. canis* and *D. immitis*. No co-infections with *D. immitis* were recorded.

The epizootiological situation with respect to infections with *A. phagocytophilum* and *B. burgdorferi* s.l. in dogs varies greatly between individual provinces. The highest percentages of dogs (more than 20 %) infected with *A. phagocytophilum* were noted in Lesser Poland, Silesia and Łódź Provinces, and the lowest percentages (below 5 %) in Masovia, Subcarpathia and Świętokrzyskie Provinces (see also Table 2). Lesser Poland, Silesia and Łódź Provinces have a significantly (in a descriptive manner) higher prevalence rate than the overall with respect to *A. phagocytophilum*, while Masovia, Subcarpathia, Świętokrzyskie and Lublin Provinces have a significantly lower prevalence rate than the overall with respect to *A. phagocytophilum*. The highest prevalence of infection with *B. burgdorferi* s.l. (>10 %) was noted in dogs from Łódź Province, and the lowest prevalence (<1 %) was found, just as for *A. phagocytophilum*, in Masovia Province. These differences in prevalence from the overall were significant in terms of a higher prevalence rate than the overall in Łódź Province and a lower prevalence rate than the overall in Masovia Province for *B. burgdorferi* s.l. Most cases of co-infection with *A. phagocytophilum* and *B. burgdorferi* s.l. were observed in Łódź Province (8.49 %), while in Masovia, Subcarpathia and Podlaskie Provinces no such cases were found (see also Table 2). The higher prevalence rate than the overall in Łódź Province is significant for co-infection with *A. phagocytophilum* and *B. burgdorferi* s.l. The few dogs positive for *E. canis* and *D. immitis* were distributed throughout the country and no clear regional focus was found.

## Discussion

The main vector of *A. phagocytophilum* and *B. burgdorferi* s.l. in Poland is the castor bean tick, *I. ricinus*. It is distributed throughout Poland, so that there is a clear potential for transmission of these two pathogens all over the country. Furthermore, both pathogens occur in dogs, can cause clinical disease in dogs, although for *Borrelia* so far only pathogenicity of the genospecies *B. burgdorferi* s.s. has been proven for dogs (reviewed by Krupka and Straubinger 2010), and both also have a zoonotic character. In several studies, mainly concentrating on man and the vector tick, the prevalence of both pathogens has been confirmed in Poland.

Most human cases of borreliosis were originally recorded in the north-eastern part of the country, but there have since also been reports of the pathogen from the north-western region in ticks (Skotarczak et al. 2002, 2003; Skotarczak 2000; Wodecka and Skotarczak 2000) and of pathogen DNA also in dogs (Skotarczak and Wodecka 2003, 2005). One study from Poland furthermore confirmed the role of the genospecies *B. burgdorferi* s.s. in canine borreliosis (Wodecka et al. 2009). Vector ticks carrying *A. phagocytophilum* have been recorded across the country (e.g., from southern (Lesser Poland) (Asman et al. 2013) to central (Warsaw) (Zygner et al. 2008) and northern Poland (Pomerania) (Stańczak et al. 2004)). Dogs mainly from the north-western region have been reported to be *A. phagocytophilum*-positive (Skotarczak et al. 2004; Rymaszewska and Adamska 2011), even though Skotarczak and colleagues (2004) presumed that the low prevalence data (PCR-based method) recorded in dogs in that area might indicate that the domestic dog is not a reservoir for *Anaplasma* in the tested region. Nevertheless, in a large German canine seroprevalence study by Krupka and colleagues (2007), some of the highest prevalences for *A. phagocytophilum* in dogs were detected in the German postcode regions 0 and 1 (23.1 % and 25.8 %), representing the German districts bordering Poland and the Czech Republic, and thus may also mirror a focus of *A. phagocytophilum* in dogs in that region covering north-west Poland and north-east Germany. Based on the data for ticks, a more nationwide exposure seems to occur for *A. phagocytophilum*, in contrast to an apparently more northerly distribution of the borreliosis pathogen.

The prevalence data from the present study could not confirm a more northerly occurrence of dogs positive for *B. burgdorferi* s.l., but showed by far the highest prevalence in Łódź Province (11.3 %), followed by Opole, Kuyavia-Pomerania, Silesia and Lower Silesia Provinces, nearly all located in the south-west of Poland (apart from Kuyavia-Pomerania). A nationwide occurrence of *A. phagocytophilum* in the tested dog population was confirmed, with the lowest prevalences in the north, north-east, central-east and south-east (Pomerania, Warmia-Masuria, Podlaskie, Masovia, Lublin, Świętokrzyskie and Subcarpathia Provinces), regions which in part make up the areas which originally had the highest occurrence of borreliosis. The highest prevalences were recorded in the central and southern regions.

Regarding *E. canis* and *D. immitis* seropositivity, the few dogs with a positive reaction (*n* = 13, or 12 after one potentially *D. immitis*/*A. vasorum* cross-reacting dog had been discounted) are distributed throughout the country, so that no clear focus can be described. The fact that so far *E. canis* has not been reported autochthonously in dogs in Poland, *D. immitis* has only been published with one questionable autochthonous case in Poland without molecular confirmation (Światalska and Demiaszkiewicz 2012), the dogs in the study that were positive for *D. immitis* did not show any co-infection with local agents such as *Borrelia*/*Anaplasma*, and finally that no clear regional focus could be identified, raises the question of these dogs' travel history and recent import status. Unfortunately no data were available on these aspects.

The cross-reactivity of the *D. immitis* component of some commercially available tests in *A. vasorum*-positive dogs has been documented (Schnyder and Deplazes 2012). This could have been a reason for the occurrence of *D. immitis*-positive samples in Poland, which has otherwise been negative for heartworm to date. And indeed, clarification of the results by using data available from another study, using partially the same dog population and testing for *A. vasorum* by using an antigen and an antibody ELISA (Schnyder et al. 2013), showed that one out of five dogs reacted positively in the *A. vasorum* tests, pointing to a potential cross-reaction. Examination for microfilariae would have aided discussion of the *A. vasorum* cross-reaction, as would the travel history of the dog concerned in areas endemic for *D. immitis*. But no data on these aspects were available. Nevertheless, the prevalence of 0.13 % with 4/3,094 dogs reacting positive for *D. immitis* in the study presented here is comparable to the prevalence obtained in an epidemiologically comparable situation in a large German study, where 4/3,005 dogs were *D. immitis*-positive in the SNAP® 4Dx® test (Pantchev et al. (2009b), based on the data of Krupka et al. (2007)). None of the dogs positive for *E. canis* or *D. immitis* had a co-infection with the other pathogen, i.e., *D. immitis* or *E. canis*.

The number of co-infections with *A. phagocytophilum* and *B. burgdorferi* s.l. has been the subject of several studies in both man and ticks. As the castor bean tick is the main vector for both pathogens in Poland, a co-infection could result from a dually infected tick. This dual infection has been reported for *I. ricinus* in northern Poland, e.g., with a prevalence of 8.3 % of 303 examined adult ticks (Stańczak et al. 2004). However, it could also be the result of a simultaneous or sequential infection by singly infected ticks. In man, the concurrence of *A. phagocytophilum* and *B. burgdorferi* was detected in 3.2 % in north-eastern Poland (Grzeszczuk et al. 2004) and in 4.5 % (15/334) (Zwoliński et al. 2004) and 17.5 % (11/63) (Tomasiewicz et al. 2004), both in mid-eastern Poland. One of the main concerns in co-infection is the possibility that the clinical appearance in the patient may be altered, thus potentially making diagnosis more difficult and leading to a more serious disease outcome (Krupka et al. 2007). Furthermore, one pathogen might pave the way for another. In one study, for example, seroreactivity to both *A. phagocytophilum* and *B. burgdorferi* was detected more frequently in suspected clinical cases in dogs than seroreactivity to either organism alone (Beall et al. 2008). This again should be borne in mind by veterinarians across the country, as exposure to both pathogens seems possible.

The fact that both are zoonotic pathogens indicates the need for greater involvement on the part of the public health authorities.

There are two limitations to this serological survey. First, the dogs' history of travel abroad and their import status were not recorded, so that, for the *E. canis*- and *D. immitis*-positive dogs in particular, an autochthonous character cannot be confirmed for Poland. Dogs testing positive in a specific area may have been exposed elsewhere. Second, a positive antibody test is not necessarily equivalent to the existence of the pathogen in the canine or vector population of a particular geographic region; it is only evidence of prior exposure to the corresponding pathogen at some point and some location in the dog’s history. With respect to the latter limitation, a more differentiated view needs to be taken of the detection of antibodies against C6 peptide of *B. burgdorferi* s.l.: The SNAP® 4Dx® test detected infection with *Borrelia* at the earliest on day 35 or day 49 post-infection, depending on the dog (Wagner et al. 2012). On the other hand, antibodies to C6 have been detected in the late stages of infection (>12 months) with a C6 detecting device (Wagner et al. 2012; Levy et al. 2008), and have been found to decrease significantly after specific treatment, so that at least for *Borrelia* the detection of C6 peptide might represent a more or less robust marker of infection.

Generally, the large number of dogs included and the fact that two of the pathogens, *A. phagocytophilum* and *B. burgdorferi* s.l., have also been reported in vector ticks, man or wild life in Poland several times support the conclusion that veterinarians should be aware of infection with these two pathogens potentially in all Polish provinces. Veterinarians should include these two diseases in their differential diagnosis and recommend the use of repellents along with prophylactic measures to prevent disease transmission by arthropod vectors.

In conclusion, this study represents a nationwide overview of the occurrence of important canine, but also zoonotic, pathogens in a large canine population in Poland. Dogs seropositive for *A. phagocytophilum* (12.31 % prevalence) and *B. burgdorferi* s.l. (3.75 % prevalence) were detected in all 16 Polish provinces, even though the prevalence varied between the different provinces and a slightly more central/southern/mid-western focus was recorded. Nevertheless, veterinarians throughout the country should be aware that these two major canine vector-borne pathogens may occur in their practice area and exposure of their canine clients is possible.

*E. canis* and *D. immitis* were much less prevalent: 0.26 % (*E. canis*) and 0.16 % (*D. immitis*). As the travel history and import status of the positive dogs were not available, an autochthonous character of the latter two pathogens cannot be confirmed.

Co-infections with *A. phagocytophilum* and *B. burgdorferi* s.l. were recorded in 1.71 % of all examined dogs, and one dog was even infected with a third pathogen, *E. canis*.
